# Rifampicin mono-resistant tuberculosis in France: a 2005–2010 retrospective cohort analysis

**DOI:** 10.1186/1471-2334-14-18

**Published:** 2014-01-10

**Authors:** Vanina Meyssonnier, Thuy Van Bui, Nicolas Veziris, Vincent Jarlier, Jérôme Robert

**Affiliations:** 1 Sorbonne Universités, UPMC Univ Paris 06, U1135, Centre for Immunology and Microbial Infections, team 13, F-75013 Paris, France; 2 Current address of Vanina Meyssonnier: Internal Medicine Department Groupe Hospitalier Diaconesses Croix Saint-Simon, Paris, France; 3 INSERM, U1135, Centre for Immunology and Microbial Infections, team 13, F-75013 Paris, France; 4 APHP, Centre National de Référence de Mycobactéries et de la Résistance des Mycobactéries aux Antituberculeux, Bactériologie-Hygiène, Hôpitaux Universitaires Pitié Salpêtrière – Charles Foix, F-75013 Paris, France; 5 Bactériologie-Hygiène, Faculté de Médecine Pierre et Marie Curie, Site Pitié-Salpêtrière, 91 Bd de l’hôpital, 75634 Paris, Cedex 13, France

**Keywords:** Rifampicin, *Mycobacterium tuberculosis*, Monoresistance, Management

## Abstract

**Background:**

Rifampicin resistance is a risk factor for poor outcome in tuberculosis. Therefore, we sought to describe the characteristics and management of Rifampicin monoresistant (RMR) tuberculosis (TB) in France.

**Methods:**

We conducted a retrospective cohort analysis in 2012 on RMR TB patients diagnosed in France between 2005 and 2010 by using a national laboratory network. A standardized questionnaire was used to collect basic demographic data, region of birth, history of TB, HIV-coinfection, alcohol use, and antituberculosis treatment. Outcome was assessed after at least 18 months of follow-up.

**Results:**

A total of 39 patients with RMR TB were reported (0.12% of all TB cases). Overall, 19 (49%) had a previous history of treatment, 9 (23%) were HIV-coinfected, and 24 (62%) were smear-positive. Patient with secondary RMR were more likely to have alcohol abuse (P = 0.04) and HIV-coinfection (p = 0.04). Treatment outcome could be assessed for 30 patients, the nine others being dead or lost to follow-up. A total of 20 (67%) of the 30 assessed were cured, 3 (10%) died, 3 (10%) relapsed, and 4 (13%) were lost to follow up. Four (13%) received less than 6 months of treatment, 3 did not have any modification of the standardized regimen, 13 (43%) received fluoroquinolones, 4 (13%) aminoglycosides, and 8 (26%) a combination of both.

**Conclusions:**

RMR TB is a rare disease in France, and its management was heterogeneous. The lack of treatment standardization may be a consequence of low expertise and may lead to the unsatisfactory low success rate.

## Background

The main risk factor for antituberculosis drug resistance is a previous treatment by antituberculosis drugs, and acquired resistance is a direct and almost immediate indicator of tuberculosis (TB) control program performance [1,2]. Acquisition of drug resistance by *M. tuberculosis* is a stepwise process because no single biochemical mechanism leads to cross-resistance amongst first line drugs. Therefore, multidrug resistance, i.e. resistance to at least isoniazid and rifampicin, arises after acquisition of either isoniazid or rifampicin resistance followed by acquisition of resistance to the companion drug. Isoniazid-resistant, rifampicin-susceptible strains are quite common. Indeed, prevalence of isoniazid mono-resistance is around 5% among new TB cases in France and in most Western Europe, and is by far more frequent in previously treated cases [3]. On the opposite, rifampicin mono-resistance (RMR) is very infrequent, and accounts for only a few cases each year in most countries. RMR has been associated with a previous history of TB treatment and HIV-coinfection [4,5]. Both types of mono-resistance represent a first step toward MDR, and therefore should be managed carefully [6]. While the impact of isoniazid mono-resistance on patient’s outcome has been re-analyzed recently [7-9], impact of rifampicin mono-resistance, which may be more problematic, is not well described [10]. Noteworthy, the management of patients with RMR TB is not fully standardized. International guidelines offer different options and no clinical trial have been conducted with the most recent drugs. For instance, in 2003, according to the American Thoracic Society, it is recommended to treat RMR TB by a prolonged association of three antituberculosis drugs during nine (isoniazid, pyrazinamide and streptomycin) to twelve (isoniazid, pyrazinamide, and ethambutol) months. It is also proposed to strengthen these regimens by the addition of a fluoroquinolone for patients with more extensive disease [11]. Although the treatment of RMR TB has not been specifically addressed in the last WHO guidelines, [12], it was specified in the 2008 version that RMR TB should receive a fluoroquinolone in place of rifampicin in association with isoniazid and ethambutol and addition of pyrazinamide during first 2 months for a duration of 12–18 months, with addition of an injectable in patients with extensive disease [13]. Finally, very recently, the IUATLD recommended treating RMR TB similarly as MDR TB [14]. Currently, no recommendations are available in France for RMR TB management.

In order to evaluate the magnitude of RMR in France, and to describe treatment’s regimens received and outcome, we conducted a nationwide retrospective study on RMR TB diagnosed between 2005 and 2010.

## Methods

RMR cases were reported yearly from 2005 to 2010 through a retrospective questionnaire by the national network of all microbiologists from hospital and private laboratories performing culture for mycobacteria and coordinated by the National Reference Center (NRC) [15].

A RMR strain was defined as a strain resistant to rifampicin and susceptible to isoniazid, on the basis of first-line drug susceptibility tests (DST) analysis on solid or liquid media performed by reporting laboratories. For each RMR case reported during the study period, a comprehensive questionnaire was sent for the purpose of the study to the laboratory and the clinician in charge to collect patients and disease characteristics, HIV co-infection, and prior history of treatment with anti-tuberculosis drugs. In addition, microbiological results, antituberculosis drugs regimens, compliance, and outcome (death, lost to follow-up, cure and relapse) were collected. All data were extracted from medical records and no personal identifiers were recorded. Therefore, the study did not require approval by an ethical committee nor by the electronic data protection commission. The molecular analysis of the mechanism of rifampicin resistance has been performed either locally or by the NRC by using the GenoType® MTBDR or MTBDR plus assays (HainLifeScience GmbH, Nehren, Germany) or in home sequencing methods [16]. Because it is a retrospective study, strains were not available for complementary analysis.

Duration of treatment was divided into four categories: < 6 months, 6 to < 9 months, 9 to 12 months and more than 12 months. All quinolones (ofloxacin, ciprofloxacin, moxifloxacin, and levofloxacin) were grouped into a single category. All aminoglycosides (amikacin and streptomycin) were also grouped into a single category.

Only those patients treated for more than one month before outcome were included in the outcome analysis. Kaplan-Meier analysis was performed to analyze the cumulative probability of unfavorable outcome, i.e. failure, in new and previously treated patients by using time from TB treatment initiation to the last medical visit. Patients with unknown outcome before the end of the programmed treatment duration, i.e. lost to follow-up, those with relapse, and deceased patients were considered as failure. Therefore, no patient was censored. Categorical variables were compared by using the χ^2^ test. *P*-values are two-tailed and p < 0.05 was considered statistically significant. Data were analyzed by using Stata 11 (Stata Corp, College Station, TX, USA).

## Results

A total of 49 TB patients with RMR were reported to the network between 2005 and 2010. Among all reported cases, 6 were excluded because of missing data precluding any analysis and 4 because of duplicate declaration. Consequently, 39 cases of RMR tuberculosis were included. By definition, all strains were resistant to rifampicin and susceptible to isoniazid. Results of molecular analysis of rifampicin resistance were available for 21 of the 39 RMR strains. The mutations identified in *rpoB* were Ser531Leu (n = 9), His526Tyr (n = 4), His526Asp (n = 2), Leu533Pro (n = 2) and Asp516Val (n = 1). In addition, 3 strains had non-identified mutations (no hybridization with the wild-type and mutation probes), and therefore were considered as having a mutation not identified by the mutations probes. No additional molecular analysis was performed on the three latter strains. For the 18 remaining strains, molecular analysis was not performed.

The total number of RMR TB reported each year during the study period varied between 3 and 11 cases, accounting for a mean annual RMR rate of 0,12% among all culture-positive TB diagnosed by the network. The 39 cases were reported by a total of 27 laboratories from 8 of 22 metropolitan regions and from two out of four overseas French departments.

Among all patients, 20 (51%) had no recognized history of TB treatment, and 19 (49%) reported a prior history of treatment (Table 1). The previous antituberculosis regimens could be fully assessed in only 12 of the 19 previously treated patients. All 12 former patients had received rifampicin-containing regimens, and 8 completed their treatment. Two patients, who were homeless and alcohol addicts, stopped the treatment by their own and went back to hospital because of clinical relapse; the two latter were opposed to any care. Of interest, 10 of the 12 patients reported frequent treatment interruptions.

**Table 1:** Characteristics of the 39 patients with rifampicin monoresistant tuberculosis reported in France between 2005 and 2010

**Characteristic**	**Total**	**History of treatment**		**P value**
	**N(%)**	**No N(%)**	**Yes N(%)**	
Total patients	39 (100)	20 (100)	19 (100)	
Male	19 (49)	8 (45)	11 (58)	0.63
Age median [IQR]	43 [29–58]	38.5 [24.5–58]	44 [36–58]	
≥ 40 years old	21 (54)	10 (50)	11 (58)	0.62
Foreign born patients	18 (46)	12 (60)	6 (31)	0.07
If yes, year of arrival in France:				
< 5 years	10	8	2	
5–10 years	4	2	2	
≥ 19 years	3	1	2	
NA	1	1		
Travel ≤ 2 years before diagnosis:				
Yes	10 (26)	7 (35)	3 (16)	0.38
No	25 (64)	11 (55)	14 (74)	
NA	4	2	2	
Social disadvantage	9 (23)	3 (15)	6 (30)	0.12
Collectivity	5 (13)	3 (15)	2 (10)	0.31
History of tuberculosis before diagnosis:	20 (51)	1* (5)	19 (100)	
< 5 years	15		15	
> 20 years	3	1*	2	
NA	2		2	
History of:				
-Incarceration	0	0	0	-
-Intravenous drug user	3 (8)	1 (5)	2 (10.5)	0.40
-Tuberculosis exposure	5 (13)	4 (20)	1 (5)	0.38
-Alcohol use	9 (23)	2 (10)	7 (37)	0.04
-Immunosuppression	4 (10)	3 (15)	1 (5)	0.32
HIV co-infection	9 (23)	2 (10)	7 (37)	0.04
Site of tuberculosis:				
-Pulmonary	24 (61.5)	12 (60)	12 (63)	0.94
-Extra-pulmonary	8 (20.5)	4 (20)	4 (21)	
-Pulmonary and extra-pulmonary	7 (18)	4 (20)	3 (16)	
Smear-positive	24 (62%)	7 (35%)	17 (89%)	<0.001

IQR: inter-quartile range; NA: not available.

*: This patient did not receive any treatment.

Among all cases, 19 (49%) were male, with a median age of 43 years (interquartile range: 29–58), 18 (46%) were born outside of France. Among patients with no history of treatment, 12 (60%) were born outside of France as compared to 6 (31%) among those with prior treatment history (*P* = 0.07).

Fourteen (36%) were living in collectivity or had a precarious social situation. Four had an immune-compromising disease (diabetes, corticosteroid therapy). Of note, 5 (13%) had contacts with relatives (grand-mother, wife, children) treated for TB, including two treated for RMR TB. Genotyping, which confirmed cross-transmission, was performed for only one couple of cases.

HIV co-infection and alcohol addiction were significantly more frequent in previously treated patients (37% and 37%, respectively) than among patients with no history of treatment (10%, *P* = 0.04; and 10%, *P* = 0.01, respectively).

A majority of patients (61.5%) had pulmonary TB, and this proportion was not significantly different according to HIV status. Sputum smear-positive TB was significantly more frequent among previously treated patients than among others (89% vs 35%, *P* < 0.001). All but one, were infected by *Mycobacterium tuberculosis* strains, and the remaining patient was infected by *M. bovis*. Overall, one strain was resistant to ethambutol, and two were resistant to streptomycin. Susceptibility tests results to quinolones and amikacin have been performed at the NRC for 20 (51%) strains, among which 2 were resistant to quinolones (the *M. bovis* strain from a patient with secondary RMR, and one from a patient with primary RMR), and none to amikacin.

Data about treatment and outcome were not available for three patients. In addition, two patients were lost to follow-up or died before treatment initiation, three were lost to follow-up during the first month of treatment course and one died before availability of susceptibility tests. Finally, treatment outcome was assessed for 30 of the 39 patients (Table 2).

**Table 2:** Characteristics of treatment regimens of the 30 rifampicin monoresistant tuberculosis patients included in the outcome analysis by outcome and for those with extrapulmonary tuberculosis

**Treatment characteristic**	**Total patients (n = 30)**	**Outcome**				**Extrapulmonary tuberculosis° n = 10**
		**Recovery (n = 20)**	**Lost to follow-up (n = 4)**	**Dead (n = 3)**	**Relapse (n = 3)**	
Total duration of treatment						
< 6 months	4 (13%)		1	2_1_	1_1_	2
6 to <9 months	2 (7%)	1		1_1_		0
9–12 months	13 (44%)	9_2_	2		2_1_	7
13–24 months	11 (37%)	10_2_	1			1
Total FQuinolones (no AG)	13 (43%)	7 (35%)	1 (25%)	3(100%)	2 (66%)	6 (60%)
1–3 months	3		1	3_2_		2
4–6 months	1					0
> 6 months	9	7			2_1_	4
Total AG (no FQuinolone)	4 (13%)	3 (15%)	1 (25%)	0	0	1 (10%)
1–3 months	3	2	1			1
> 3 months	1	1				0
FQuinolones and AG*	8 (26%)	6 (30%)	1 (25%)	0	1 (33%)	1 (10%)
Duration of AG						
1–3 months	6	5_3_			1_1_	0
> 3 months	2	1	1			1
Duration of FQuinolones						
1–3 months	2	2_1_				1
4–6 months	1		1			0
> 6 months	5	4_1_			1_1_	0

Numbers in indices indicate the number of HIV co-infected patients for the category, if any.

FQuinolones = any fluoroquinolone, AG = any aminoglycoside.

*Duration of the combination of at least one month.

°: 5 pulmonary and extrapulmonary tuberculosis and 5 with only extrapulmonary tuberculosis.

At the time of TB diagnosis, and before susceptibility tests results, 25 (83%) out of the 30 patients received rifampicin-containing regimens. The five remaining patients did not receive rifampicin because of suspected resistance according to previous treatment history or availability of first line susceptibility tests from other countries or contact. Among them, 3 received a combination of moxifloxacin and aminoglycoside with the three other first line drugs (one receive isoniazid only after availability of susceptibility tests), one moxifloxacin combined to isoniazid and pyrazinamide, and one amikacine along with other first line drugs.

Four (13%) patients received less than 6 months of treatment, including two because of death at three and five months of treatment, and two that stopped their treatment after 3 months and were lost to follow-up, including one which eventually relapsed the following year. For the 26 remaining patients, the duration of treatment spanned from 8 months (n = 2, 7%), 9 to 12 months (n = 13, 44%) patients, and 11 to 24 months (n = 11, 37%). Overall, good adherence to TB treatment, i.e. no interruption of > 1 week, was reported in 20 (74%) patients.

When susceptibility tests results to first line drugs were available, 3 patients did not have any modification of the rifampicin-containing regimen. Among these three patients, two were considered as cured after 9 months of treatment (two months of standard four-drug regimen and 7 months of rifampicin and isoniazid) with more than 2-year follow-up. The third patient, who had a Kaposi sarcoma related to HIV-coinfection, died after 9 months of standard treatment.

Thirteen (43%) patients received fluoroquinolone-containing regimens without aminoglycoside, 4 (13%) received amikacin-containing regimens without fluoroquinolone, and 8 (26%) received regimens containing both fluoroquinolones and amikacin. Among the 21 patients who received at least one month of fluroquinolone, 15 (71%) received moxifloxacin. Patients with extrapulmonary TB (n = 10), and those with a history of antituberculosis treatment (n = 14) were not more likely to receive fluoroquinolones (70% and 79%, respectively) than patients with only pulmonary TB (n = 20, 70%; P = 1.0) or with no history of antituberculosis treatment (n = 16, 63%; P = 0.34). On the opposite, the 18 with smear-positive TB were more likely to receive fluoroquinolones than the 12 patients with smear-negative TB (89% versus 30%, respectively; P = 0.003).

**Figure 1:**
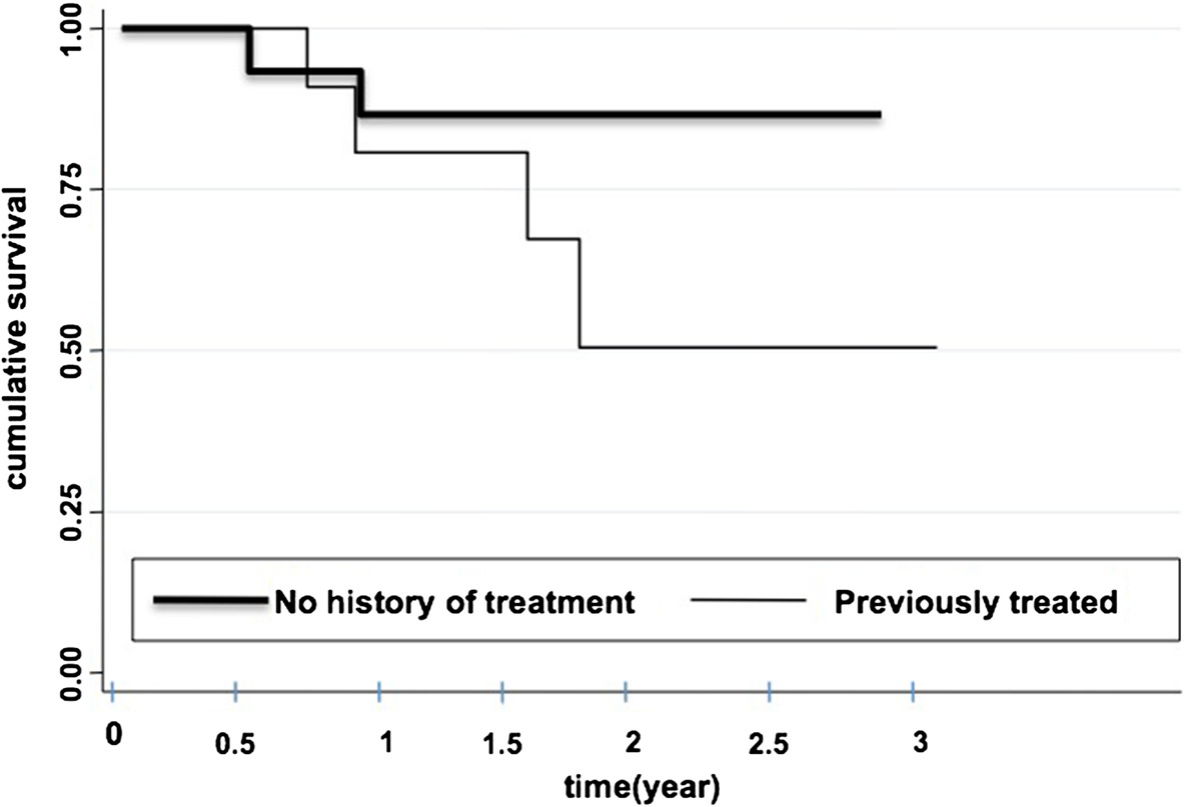
Patients’ survival (Kaplan-Meier analysis) according to previous history of treatment.

A total of 20 patients (67% of the patients with follow-up, but 51% of all RMR patients) were considered as cured or completed treatment with clinically favorable outcome at the last registered visit (18 months of median follow-up after onset of TB treatment), including 13 who received fluoroquinolones, 9 who received aminoglycosides, and 19 with a duration of treatment of at least 9 months. Outcome was considered as unfavorable for the other ten (33%) followed patients (3 relapses, 4 lost of follow-up, and 3 deaths). The outcome was less favorable for those with a previous history of treatment (50%) than for new patients (19%) (Figure 1), but the difference was not statistically significant (p = 0.14, log-rank test). No statistically significant difference was observed according to HIV status (data not shown).

Patients treated with fluoroquinolones for at least 6 months were slightly more likely to have favorable outcome (11 out of 14 cases, 78%) than others (9 out of 16 cases, 56%) but the difference was not statistically significant (p = 0.20).

## Discussion

The impact and the management of RMR TB have not been deeply studied as compared to MDR TB, which is well known to be associated with poor TB outcome [1]. We evaluated the characteristics and the management of RMR TB in France over a 6-year study period. We confirmed that RMR is a rare event (0.12% of all culture-positive cases). We report a high proportion of RMR among new patients. Finally, we showed that RMR TB management was quite heterogeneous in France during the study period, with neither standardized drug regimen nor duration of treatment, and that the outcome was not very satisfactory.

In France, RMR TB is a rare event. The overall observed proportion of 0.12% is similar to those reported in countries with similar epidemiological profile of TB such as Western Europe. In 2010, RMR TB accounted for 0.3%, 0.3%, and 0.1% of primary TB cases, and 1.9%, 0%, and 0.2% of secondary TB cases in Germany, United Kingdom, and Poland, respectively [17]. However, RMR seems to be higher in high incidence countries such as Zambia in 2006 where it reached 1.3% among new cases, and 3.2% among previously treated cases [18]. There is an alarming report from South Africa regarding a rise in RMR TB among HIV-coinfected persons [19]. It has been previously reported that RMR was associated to HIV-coinfection [5,20-23]. Our report and the one from South Africa confirm this association [19]. Indeed, the 23% of HIV-positive patients in the current study contrast with the one reported among all patients in France (10%) and among MDR TB cases reported in 2009 (9%) [5,24]. The reasons for such an association have not been fully clarified. Exposure to RMR sources in health care facilities or at home may explain primary RMR. Cross transmission has been demonstrated in 13% of the cases in the USA, and family exposure is likely or confirmed in two (10%) of the 20 primary cases in our study [4].

Alcohol abuse has been already associated with RMR TB [19]. In the present study, it has been linked to RMR in previously treated cases in univariate analysis. It is likely that it is an indirect measure of unstable social background and bad compliance leading to selection of resistant mutant during treatment [25-27]. Interference with pharmacokinetics of antituberculosis drugs has been suggested but, to date, this has not been confirmed [28].

We report a high heterogeneity in RMR TB management in France. Many reasons may lead to this worrying situation. First, it is a rare disease and therefore, it is difficult to build an expertise for most clinicians. To reinforce this issue, cases were disseminated throughout the French territory, and no single referral team has been identified. Second, guidelines regarding RMR TB available at the time of the study offer many treatment options making standardization more difficult [11-14]. However, the IUATLD guidelines published in 2013 after the completion of our study recommend treating RMR TB similarly as MDR TB [14]. A large proportion of strains were not sent to an expert laboratory for testing against second line drugs making the choice of treatment much more random although very few additional resistance were observed among tested strains. It has been proven that treatment of RMR TB can be very successful if aminoglycosides and fluoroquinolones are used, along with a prolonged treatment duration [29]. Finally new drugs recently approved to treat MDR TB may improve the outcome. Therefore, we suggest that RMR TB management in France follows the same principles as those currently accepted as good practices for the management of MDR TB [12]. We implemented such a strategy after the dramatic observation of a low success rate in the treatment of MDR TB cases in the 1990s [30,31]. This strategy was focused only on MDR TB but overlooked RMR TB. Laboratories should be aware about the requirement to confirm rifampicin resistance and isoniazid susceptibility, as well as to determine susceptibility to second-line drugs in case of rifampicin resistance. In addition, the treatment standardization by referring to an expert team, as recommended for the management of MDR TB, should improve the RMR TB outcome. Finally, it is likely that the use of rapid molecular evaluation of rifampicin resistance has spread in France since the start of the study in 2005. This should be confirmed and the interest of such test should be reemphasized [12].

Two (10%) of the 20 strains with *rpoB* mutations had a Leu533Pro genotype that confers a low-level of rifampicin resistance (minimal inhibitory concentration, MIC of 0.5 mg/L) [32]. The peak to serum concentration of rifampicin being close to 10 mg/L, rifampicin could retain some activity against such strains [33]. The recent interest of the moxifloxacin use against strains with low-level of fluoroquinolone resistance reinforces this hypothesis [34]. Consequently, we suggest that MIC determination should be performed for all strains with a Leu533Pro genotype or for strains with mutations with unknown impact on the rifampicin MIC level.

Our study is an observational study with a limited number of cases. Therefore, it is lacking of power to draw definite conclusion regarding the interest of fluoroquinolones or aminoglycosides in place of rifampicin in combination with isoniazid for the treatment of RMR TB as well as the role of characteristics such as HIV coinfection of previous treatment history. The interest fluoroquinolones and aminoglycosides has been reported for MDR TB [27,28,35]. Because the present study is observational, the choice the two latter drugs, or of the duration of the treatment may be linked to variables not fully assessed such as severity of illness or co-morbidities. Consequently, we did not attempt to identify treatment regimens significantly associated with a favorable outcome because of potential biases. Pooling data from different countries and different observational studies will make it easier to evaluate the relative interest of each drug and of the optimal duration of the treatment as it has been recently reported for MDR TB [36].

## Conclusion

In conclusion, the present study alerts on the heterogeneity of the management of RMR TB in a low incidence country. Despite its paucity, RMR should be carefully managed because it has a worse outcome than pan-susceptible TB, and it is a first step toward MDR TB. Efforts should be driven on comprehensive drug susceptibility testing, and standardized management. The implementation of a prospective cohort with a register will help in the evaluation of the program.

## Competing interests

The authors declare no competing interest.

## Authors’ contributions

VM designed the study, collected and analysed and interpreted the data, and draft the manuscript. TVB participated in the design of the study, data collection and interpretation of data. NV participated in data interpretation and revised the manuscript. VJ participated in data interpretation and revised the manuscript. JR conceived the study, and participated in its design, coordination and in data analysis, data interpretation and draft the manuscript. All authors read and approved the final manuscript.

## Pre-publication history

The pre-publication history for this paper can be accessed here:

http://www.biomedcentral.com/1471-2334/14/18/prepub
